# Excellent Anode Performance of N-, P-, and As-Doped Graphdiynes for Lithium-Ion Batteries

**DOI:** 10.1021/acsomega.3c04605

**Published:** 2023-09-13

**Authors:** Baoyan Li

**Affiliations:** Department of Chemical Engineering, University College London, LondonWC1E 7JE,U.K.

## Abstract

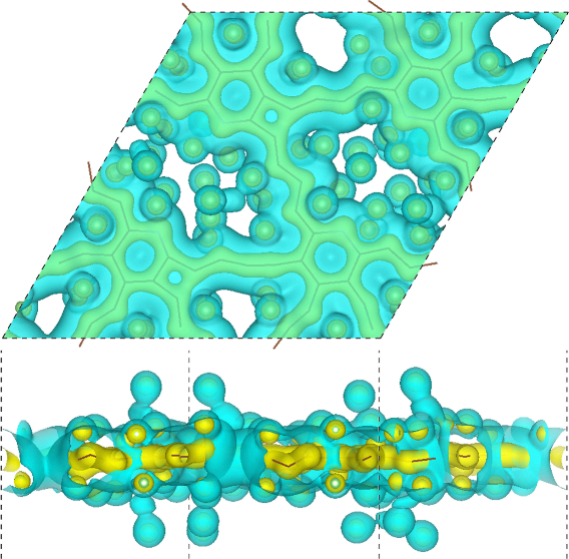

Recently, graphdiyne (GDY) as a two-dimensional planar carbon allotrope has received significant research attention in the fields of rechargeable batteries, catalysis, biomedicine, and so forth. However, the theoretical capacity of a perfect GDY anode is only 744 mA h/g in the configuration of LiC_3_, encouraging further efforts to increase the capacity. In this study, we explore the anode performance of N-, P-, and As-doped GDYs by using first-principles calculations. Ab initio molecular dynamics simulations show that the doped GDYs can remain stable at 1000 K, indicating good thermal stability. With the loss of part acetylenic linkages, the rhomboid-like pores produce more Li sites, and the theoretical capacities reach 2209, 2031, and 1681 mA h/g for the N-, P-, and As-doped GDYs, respectively. In addition, the transition-state calculations indicate that the Li diffusion barriers of the three doped GDYs are similar to the perfect GDY. This study demonstrates that doping is an effective strategy to improve the anode performance of GDY.

## Introduction

Over the past 30 years, great efforts have been devoted to seeking new carbon allotropes, and the famous graphene,^1^ carbon nanotubes,^2^ and fullerenes^3^ have been added to this family. Graphyne as a new carbon allotrope was first predicted in 1987,^4^ which contains benzene rings and acetylenic bonds with sp^2^- and sp-hybridized carbon atoms. Among various graphyne structures, graphdiyne (GDY) was first synthesized in 2010^5^ through the cross-linking reaction of hexaethynylbenzene on a copper surface. Recently, GDYs have exhibited extraordinary intrinsic properties including natural band gap, good conductivity, and excellent fabricability due to the conjugated acetylenic bonds and the numerous in-plane cavities.^6,7^ The fascinating properties inspire the attempts to apply GDYs in catalysis,^8−10^ electrochemical energy storage,^11−15^ biomedicine,^16^ thermoelectric and photoelectric conversions,^17−19^ hydrogen storage,^20,21^ water purification,^22^ and gas separation.^23^

The benzene rings connected through acetylenic linkages in GDYs provide abundant ion-storage sites and diffusion passageways, which facilitate the anode applications for rechargeable batteries.^7^ However, the theoretical capacity of the GDY anode is only 744 mA h/g for lithium-ion batteries (LIBs) in the configuration of LiC_3_,^24^ which is much lower than the capacities of alloy anodes like Si (4200 mA h/g), Ge (1568 mA h/g), and Sn (990 mA h/g) in the phase of Li_22_X_5_, where X indicates the anode element.^25,26^ Therefore, much attention has been paid to improving the capacity of the GDY anode. Lu et al.^27^ proposed a strategy of H and F positioning balanced codoped GDY, which combined the contributions of H and F elements for the Li storage, and a capacity of 2050 mA h/g at 50 mA/g was obtained with only 23% reduction after 8000 cycles. Ren et al.^28^ presented a method to drill holes in a GDY carbon skeleton by adjusting the acetylenic linkages, by which a capacity of 1550 mA h/g at 50 mA/g was achieved. Mohajeri et al.^29^ theoretically explored the Li storage on the edge oxidized GDY. They found that the oxygen groups can affect the electronic properties of GDY and further change the binding energy of the Li atom. Gao et al.^30,31^ developed cathodes with multilevel structures of GDYs, where the layer-by-layer 2D confinement effect leads to a low-strain nature. A 934 Wh/kg energy density and a long cycle life of 3000 cycles at 1 C were achieved. Shang et al.^32^ prepared N-doped GDY, and a specific capacitance of 250 F/g was obtained in the two-electrode supercapacitors assembled by the as-prepared GDYs, indicating high performance as the electrochemical electrode. However, the anode properties of N-doped GDY for LIBs were not reported. Further efforts are still desirable to optimize the GDY to satisfy the various requirements, including voltage, stability, and rate performance, for commercial applications.

First-principles density functional theory (DFT) is a powerful method to explore the low-dimensional materials for the applications of electrode^33−35^ and catalysis.^36^ The present study explores the anode performances of N-, P-, and As-doped GDYs by using first-principles calculations. After the doped structures are constructed, the thermal stability and electronic density of states are compared with the perfect GDY. Then, the Li capacities and kinetics are discussed. The capacities of N-, P-, and As-doped GDYs reach 2209, 2031, and 1681 mA h/g, much higher than the perfect GDY, and the diffusion barriers of Li on the doped GDYs are similar to that on the perfect one.

## Methods

Vienna Ab initio Simulation Package (VASP)^37,38^ was applied to conduct the first-principles calculations within the Perdew–Burke–Ernzerhof (PBE) functional^39^ to describe the exchange-correlation potential. Projected augmented wave method^40^ and pseudopotential method were used to replace the core electrons. We set a plane-wave cutoff energy of 520 eV for the DFT calculations. To test whether the N-, P, and As-doped GDYs have magnetizations, we compare the results obtained based on ISPIN = 1 and ISPIN = 2. Results show that all atoms have zero magnetization, and the free energies of the three systems are just the same as the free energies when ISPIN = 1. Therefore, we can conclude that these doped GDYs are not magnetic, and ISPIN = 1 is used for all calculations. VESTA^41^ is used for the visualization of structures and charge distributions. The *k*-point meshes were automatically generated by vaspkit^42^ with a density of 0.03. In the geometry optimization, the criteria for the energy convergence were considered as 10^–4^ eV. van der Waals interactions between the Li atom and the doped GDYs were evaluated by the DFT-D3 method of Grimme et al.^43^ including Becke–Johnson damping. Climbing-image nudge elastic band method^44^ in the VASP transition-state tools was used for the diffusion barrier of Li atoms. A 0.01 eV/Å threshold for the largest force orthogonal was set to seek the lowest diffusion path.

## Results and Discussion

**Figure 1:**
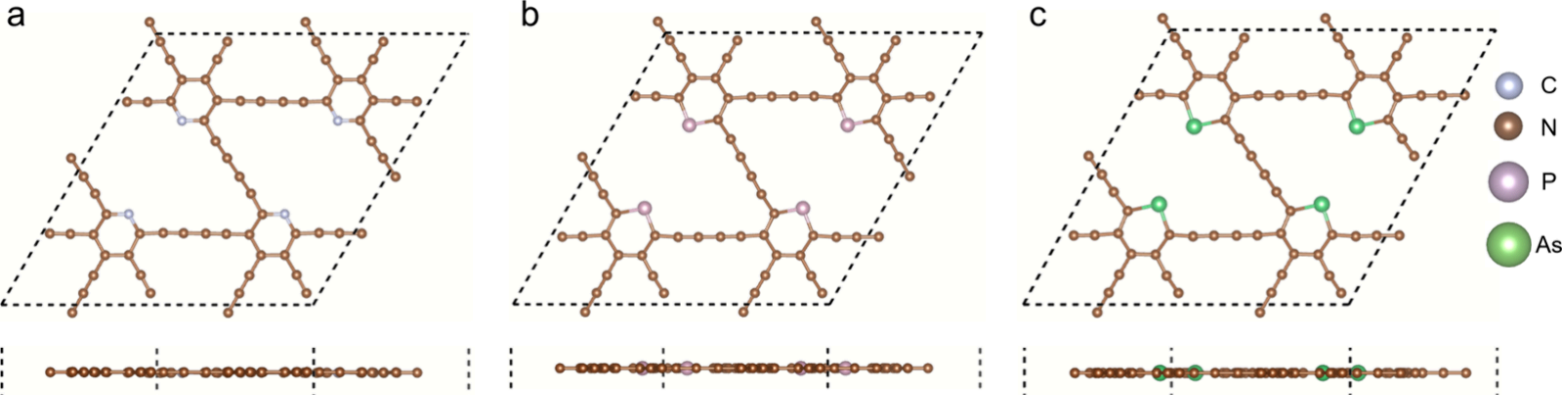
Geometry optimization of N-, P-, and As-doped GDYs, where (a), (b), and (c) show N, P, and As as the dopants, respectively.

The structures of N-, P-, and As-doped GDYs are shown in Figure 1. A 20 Å vacuum layer in the direction perpendicular to the layer is set to avoid interactions between the periodic images. The construction of the doped models is based on a 2 × 2 supercell of GDY, where a C atom in each hexatomic ring is replaced by the dopant element. The formation energy of doping can be obtained by the following relation:^45^
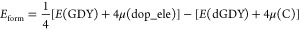
1where *E*(GDY) and *E*(dGDY) are the total energies of pristine and doped GDYs and μ(dop_ele) and μ(C) are the chemical potentials of the dopant element and carbon atoms. We obtain *E*_form_ values of N, P, and As doping into GDY as 1.508, 1.413, and 1.327 eV. The positive values indicate that the doped structures have lower formation energies and thus are feasible to synthesize.

Because of the tervalent nature of N, P, and As, the bonds of the dopant element are just saturated in the hexatomic ring with partial double bonds, and thus the acetylenic linkages connected with the dopant element will be broken away from the doped GDYs.^32^ Then, the geometries of the doped GDYs are optimized to acquire the configurations with the lowest energies. Lattice parameters of the perfect GDY are obtained to be 18.87 Å for the 2 × 2 supercell, which is close to the previous prediction of 9.48 Å for the primitive cell,^24^ indicating a good accuracy of the present calculations. Then, the lattice parameters of the doped GDYs are listed in Table 1. With the increase of the dopant element radium, the lattice parameter presents an increasing trend, that is, 18.5 Å for N-, 18.74 Å for P-, and 18.95 Å for As-doped GDYs. The N (P or As)–C bond length is positively related to the lattice parameter. Although doping changes the atom number and the lattice parameter, the main bond types and the planar structure are maintained.

**Table 1:** Lattice Parameters of 2 × 2 Supercells of the N-, P-, and As-Doped GDYs

**materials**	**lattice parameters (Å)**	**N (P or As)–C bond length (Å)**
GDY	18.87	
N-doped GDY	18.5	1.34
P-doped GDY	18.74	1.76
As-doped GDY	18.95	1.89

**Figure 2:**
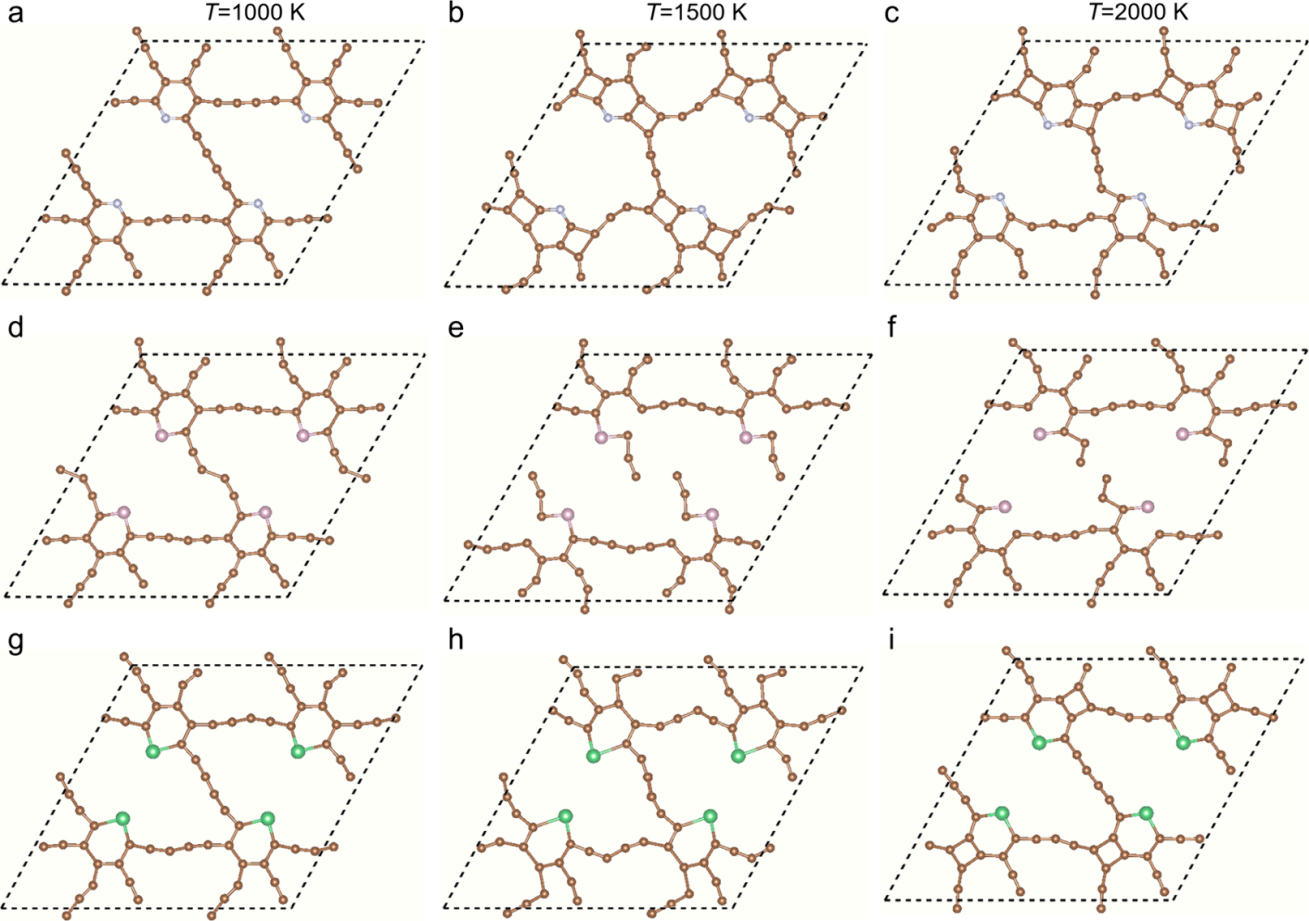
Stability of N-, P-, and As-doped GDYs at high temperatures, where (a), (b), and (c) are for N-doped GDY at 1000, 1500, and 2000 K, respectively, (d), (e), and (f) are for P-doped GDY at 1000, 1500, and 2000 K, and (g), (h), and (i) are for As-doped GDY at 1000, 1500, and 2000 K. AIMD simulations of 10 ps are carried out for each case.

The missing acetylenic linkage may decrease the stability of the doped GDYs. Ab initio molecular dynamics (AIMD) simulations are carried out at 1000, 1500, and 2000 K to evaluate the thermal stability of the doped GDYs, and the atomic snapshots are shown in Figure 2. At the temperature of 1000 K, the acetylenic linkages are slightly curved due to the thermal motion. However, there is no chemical bond broken and no new chemical bond formed, indicating good stability. Then, as the temperature increases to 1500 and 2000 K, the intense thermal movement destroys the acetylenic bonds, and some adjacent C atoms form the four-membered ring. Specifically, eight four-membered rings of C atoms in the N-doped GDY appear at 1500 K, and each includes two C atoms in the hexatomic ring and the others in the acetylenic linkage. Then, some P–C bonds are broken in the P-doped GDY as well as the acetylenic bonds at 1500 and 2000 K. Considering that the GDY nanoscroll also showed stability within 1000 K,^46^ the doping to the GDY hardly reduces the thermal stability. As LIBs generally operate around room temperature, the thermal stability of the doped GDYs is enough for the anode materials.

**Figure 3:**
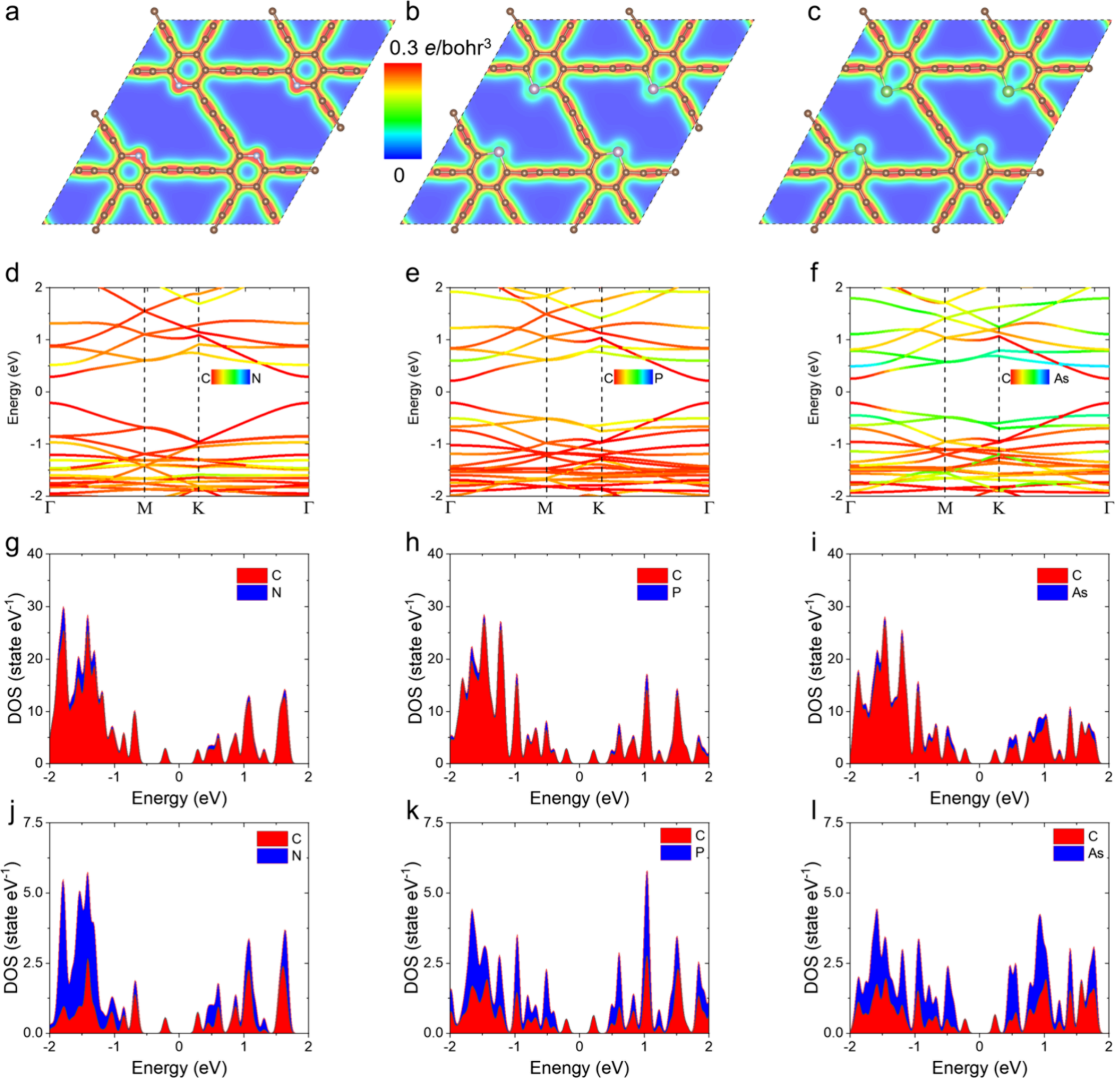
Electronic characters of N-, P-, and As-doped GDYs, where (a), (b), and (c) are the charge densities with N, P, and As as the dopants, respectively, (d), (e), and (f) are the electronic band structures with colors to represent the contribution of elements, (g), (h), and (i) are the corresponding DOSs of the dopants and all C atoms, and (j), (k), and (l) are the DOSs of dopants and the C atoms connected with the dopants.

The charge densities and electronic band structures of the doped GDYs are shown in Figure 3. The electronegativities of C, N, P, and As elements are 2.55, 3.04, 2.19, and 2.18, which can qualitatively explain the results of the charge densities. N has a higher electronegativity than C, resulting in a higher charge density around N atoms because of the charge transfer from adjacent C atoms (Figure 3a). However, for the other two doped GDYs, the charge density values around the P and As atoms are smaller than those around N atoms or C atoms. That is attributed to the charge transfer from P and As to C atoms making the electron depletion around the dopants. The band structures of the three doped GDYs (Figure 3d–f) are quite similar, although the charge densities are slightly different. Colors from red to blue are used to show the relative contributions of C and dopants to the states. The valence band tops and the conduction band bottoms for the three doped GDYs are located in the Γ point, indicating direct band gaps, which are the same as the pure GDY.^6,13,18^

As to the density of states (DOS), the band gaps of these doped GDYs are about 0.45 eV, which is very close to 0.46 eV of a GDY sheet.^6^ Therefore, it seems that the dopants hardly change the electronic conductivity. Considering the good performances of GDY anodes,^7^ the electronic conductivity in the doped GDYs is good enough for them to be anode materials. Note that the present PBE functional may underestimate the values of the band gap, and therefore, they can only be used to qualitatively understand the electronic characters of doped GDYs. To understand the bonds between C atoms and dopant elements, the DOSs of dopants and these C atoms connected with the dopants are also shown (Figures 3j–l). Good consistencies of the DOS distributions of the C atoms and dopant elements are found, indicating the covalent bond characteristics in the structures. In addition, if focusing on the energy range between −2 and −1 eV which includes most states of C atoms, most states of the N atom are in this range, while some states of P and As atoms are distributed in the energy range between −2 and 2 eV. That indicates a higher bond energy of N–C bonds than P (As)–C bonds.

The capacity and open-circuit voltage are the critical properties of an anode. The binding energy, *E*_b_, to Li and the open-circuit voltage, *V*, of the doped GDYs are defined as follows:
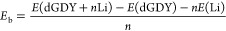
2
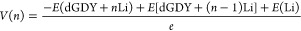
3where *E*(dGDY + *n*Li), *E*(dGDY), and *E*(Li) are the total energies of doped GDY with *n-*adsorbed Li, only doped GDY, and only a Li atom in the most stable crystal, respectively, *n* is the number of adsorbed Li atoms, and *e* is the charge quantity of an electron. Note that the reference energies of Li atoms are the ones in the bulk lattices and not free atoms in a vacuum. Obviously, a negative binding energy indicates a stable adsorption, and a lower binding energy (negative but the absolute value is high) means a stronger adsorption. If the bonding energy increases (still negative but the absolute value becomes smaller), the adsorption becomes weaker. We first test the Li capacity of the doped GDY according to the criterion of *V* larger than zero.^47^ When accommodating 64 Li atoms, the open-circuit voltages become close to zero for all three doped GDYs, and a negative *V* appears if *n* > 64 where Li atoms tend to escape from the anode and form crystals.^48^ Therefore, only the configurations of doped GDY with 64 Li atoms are discussed.

**Figure 4:**
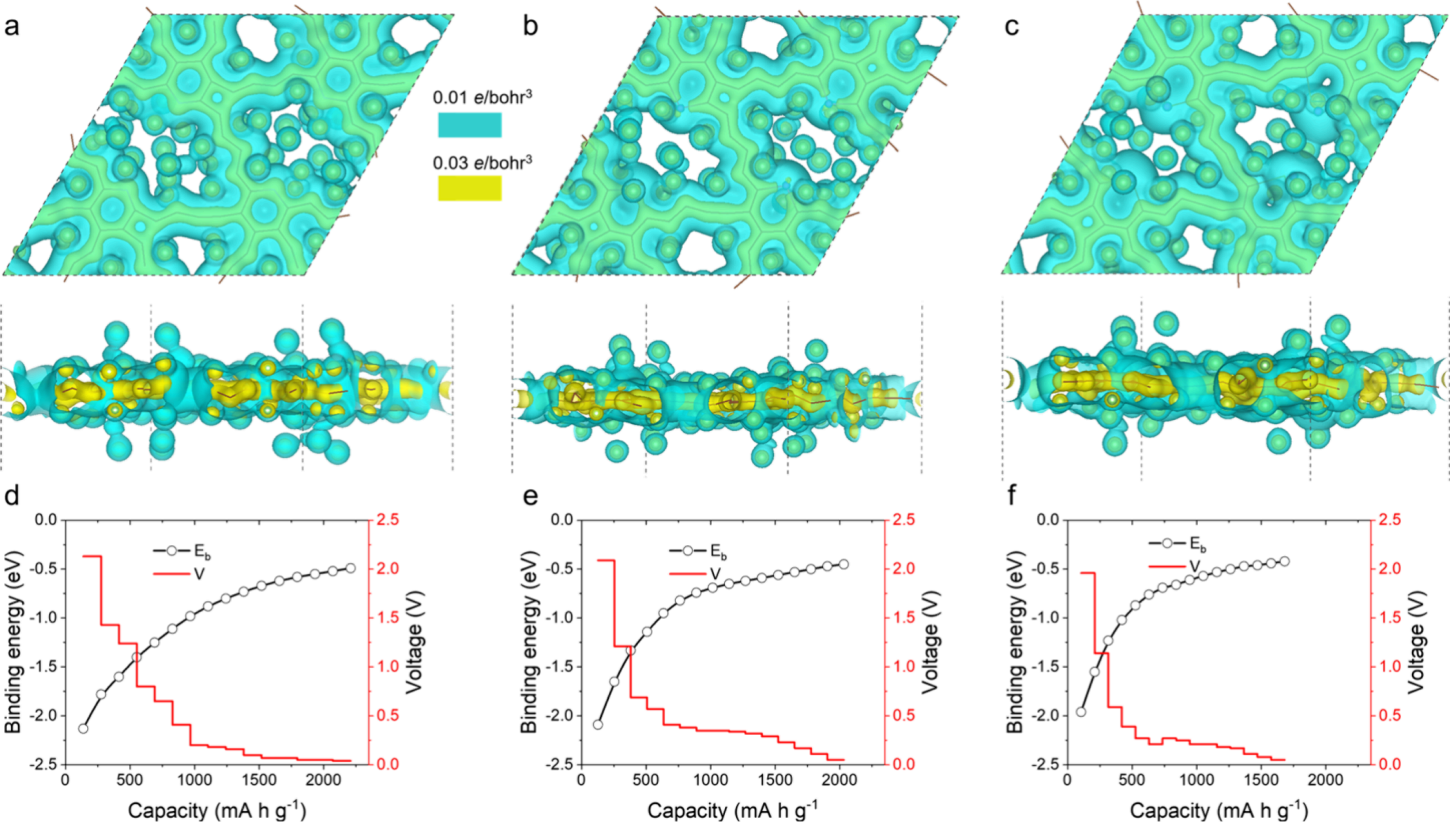
Binding energy and open-circuit voltage of the anode of LIBs, where (a–c) are the charge densities with N, P, and As as the dopants when adsorbed by 64 Li atoms and (d–f) are the binding energy of adsorbed Li atoms and the open-circuit voltage during the Li capacity variation. Yellow and blue indicate the isosurfaces with higher (0.03 e/bohr^3^) and lower (0.01 e/bohr^3^) density values.

The charge densities of the doped GDYs with Li atoms are shown in Figure 4a,b. After Li adsorption, the bonds in the doped GDYs are warped. Specifically, the acetylenic linkages become straight to serpentine, and some four-membered rings of C atoms are formed. That is because the strong attraction and charge transfer from Li atoms breaks the original symmetries of the geometry and charge. Such a deformation was also found in the triphenylene-GDY after Li adsorption,^49^ and the recoverable deformation is not expected to affect the capacity. Two charge-density isosurfaces with high and low values are displayed, where the high-value isosurface only encases the doped GDY skeleton and the low value one can represent the charge sharing between Li and doped GDY. Generally, due to the lower electronegativity of Li (0.98), the electrons turn to transfer from Li to the GDY layer, and then the induced Coulomb attractions can result in stable adsorptions of Li atoms.

With the increase of the Li number, the capacities of N-, P-, and As-doped GDYs increase to 2209, 2031, and 1681 mA h/g, respectively, which is much higher than 744 mA h/g of perfect GDY (LiC_3_).^24^ The greater ability to stably store more Li in the doped GDYs might be owing to the fact that more Li sites are produced in the large rhomboid-like pores from the loss of acetylenic linkages. Then, the binding energy of the Li atom gradually increases with the capacity due to the rising Coulomb repulsion among the Li atoms. N-doped GDY has a lower binding energy than P- and As-doped ones, meaning stronger adsorption to Li. That is owing to the higher ability of the N atom to capture the electrons of the Li atom, inducing a higher valence state of Li on the N-doped GDY and also the Coulomb attractions. The voltages of these doped GDYs decrease from about 2 to almost 0 V as the capacities approach the upper limits. The average voltages are in the range of 0.38–0.47 V, smaller than the average voltage of 0.74 V in the stacked GDY,^50^ which may be because of the stronger adsorption and lower capacity of Li in the intercalation site. In short, with the introduction of the N, P, and As atoms in the GDYs, the anode capacities were significantly enhanced.

**Figure 5:**
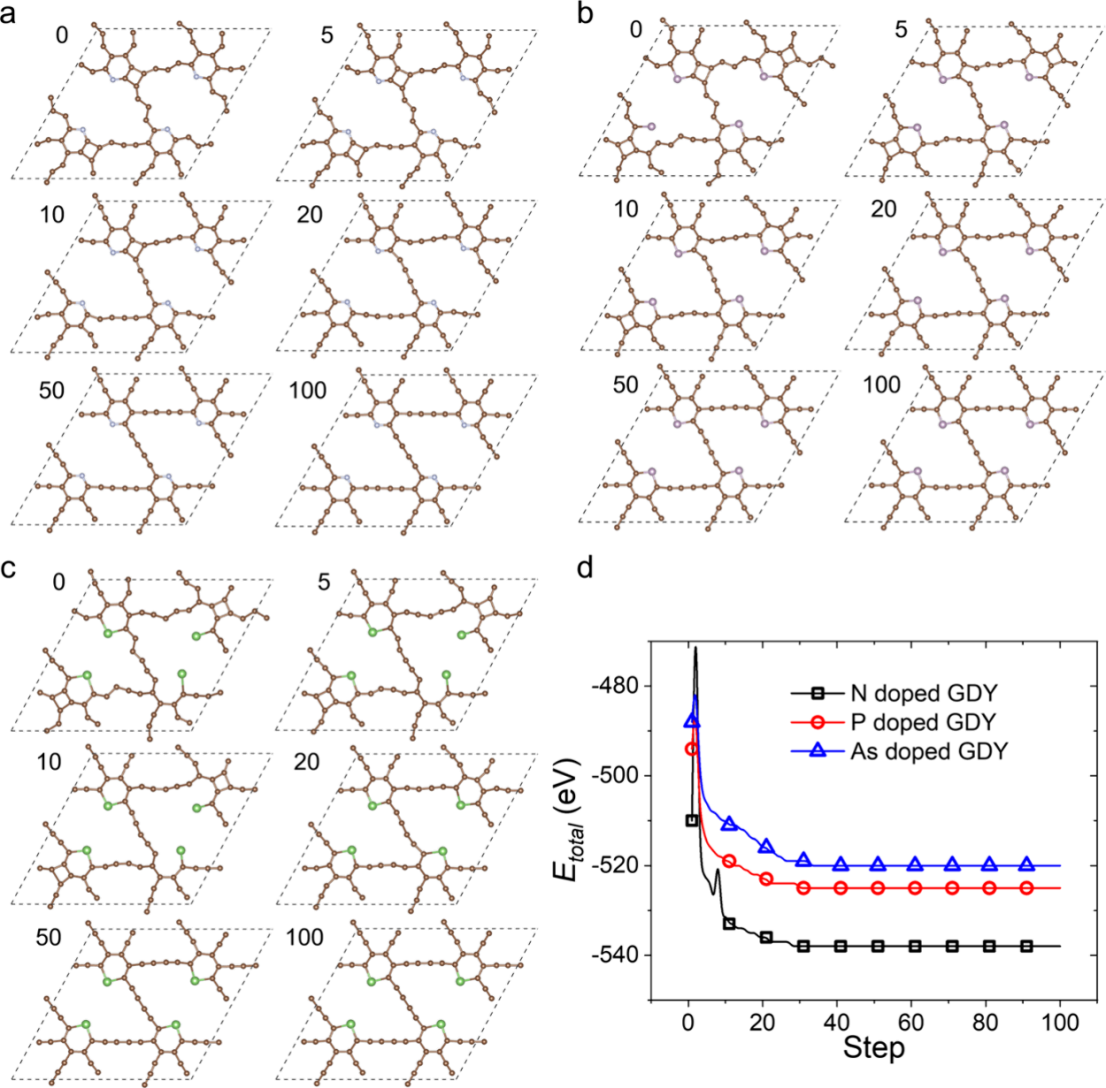
Structure recoveries of the doped GDYs after Li release, where (a), (b), and (c) are the snapshots of N-, P-, and As-doped GDYs at different steps, respectively, and (d) the total energies, *E*_total_, of the doped GDYs evolving with step.

After storing such a high amount of Li, the diyne linkages in the structure are strongly curved. Therefore, the stability of the doped GDYs after storage of Li should be carefully evaluated. Herein, we try to use the structure recoveries of the doped GDYs after Li release to judge the stability of the structure to store Li. First, all Li atoms are removed from the optimized doped GDYs with filled Li, leaving only the curved structures (see Figure 5 at 0 step). Then, the curved structures are used to perform geometry optimizations. The snapshots of N-, P-, and As-doped GDYs at 0, 5, 10, 20, 50, and 100 steps are displayed (Figures 5a–c), which can clearly show the recoveries of the structures after Li release. The total energies, which decrease at first and then become stable, also indicate the stabilities of the structures. Especially, the recoveries need only 100-step optimization, meaning a very short time might be needed for the doped GDY anode to restore to the initial structures. Therefore, the structures of the doped GDYs are restorable during the Li storage and release, and their stabilities could be reliable.

**Figure 6:**
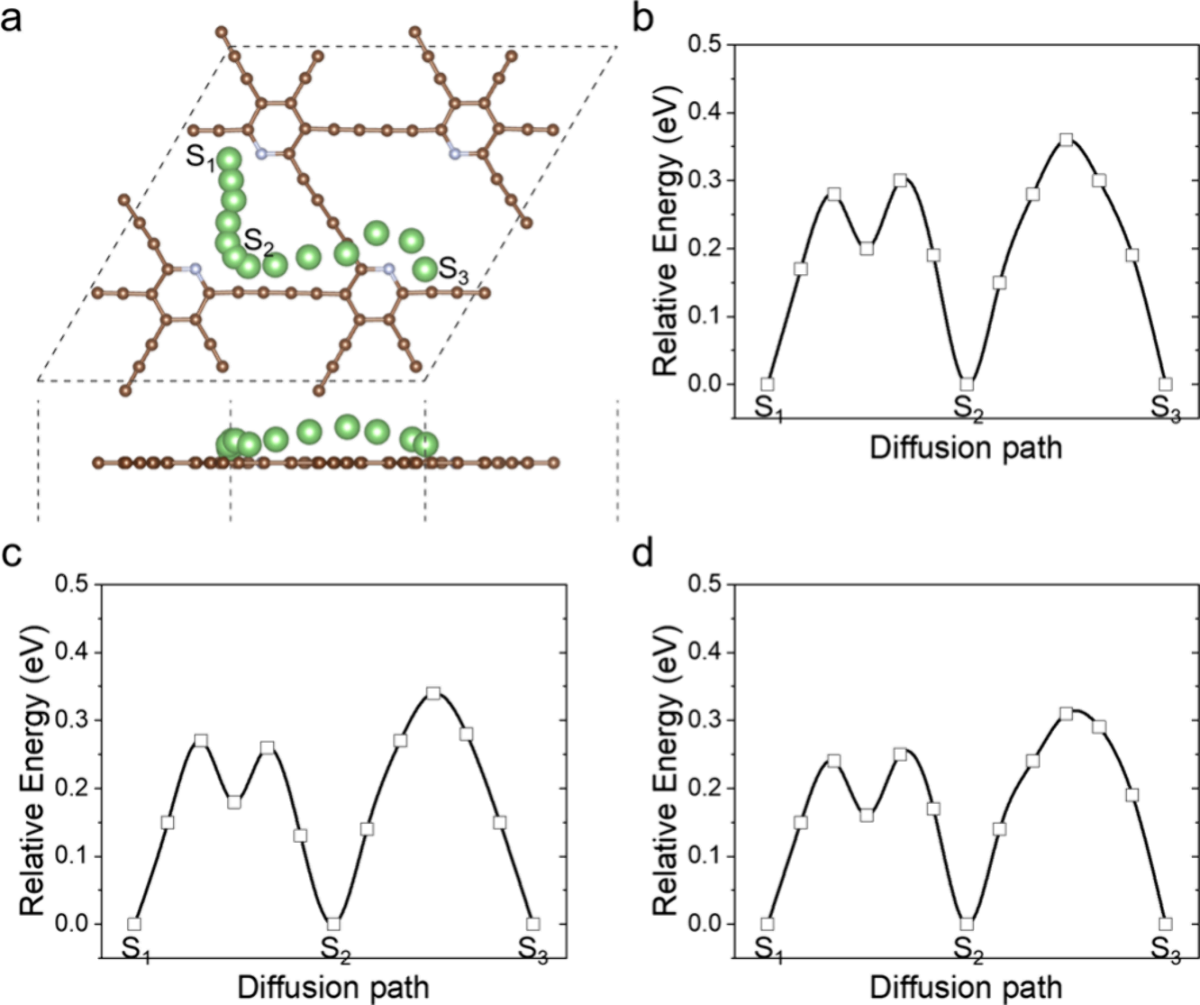
Diffusion barrier for Li atom migration, where (a) is the lowest energy path of the Li atom on the N-doped GDYs and (b–d) are the energy profiles with N, P, and As as the dopants. A, B, and C are the sites to show the diffusion path.

To analyze the kinetics of the adsorbed Li atoms, transient state calculations are carried out to obtain the energy barrier for Li atom diffusion. Figure 6a presents the diffusion path with the lowest energy of Li on the N-doped GDY. The diffusion paths on the P- and As-doped GDYs are not shown as they are similar to the N-doped case. The symbols S_1_, S_2_, and S_3_ indicate three stable sites of Li atoms, and the preset path for Li migration is from S_1_ to S_2_ and then S_3_. The energy profiles during the migration are shown in Figure 6b,c,d for N-, P-, and As-doped GDYs, respectively. From S_1_ to S_2_, the highest energies, that is, the diffusion barriers, are 0.28, 0.27, and 0.25 eV for the three doped GDYs. The decreasing trend might be derived from the declining attractions from N to As. Then, as the path from S_2_ to S_3_ passes through an acetylenic linkage, the diffusion barriers increase to 0.36, 0.34, and 0.31 eV. The diffusion barrier of Li on a monolayer GDY is 0.315–0.51 eV,^24,50^ meaning the dopants do not significantly affect the Li kinetics. Then, comparing with the 0.47–0.48 eV diffusion barrier of Li on graphene,^51^ the anodes with the doped GDYs are expected to have better rate performance than graphene.

## Conclusions

In this study, the anode performances of N-, P-, and As-doped GDYs were explored by using first-principles calculations. The doped GDYs were constructed based on a 2 × 2 supercell of GDY, where a C atom in each hexatomic ring was replaced by the dopant elements, and two acetylenic linkages connecting with the dopant were removed due to the tervalent nature of N, P, and As. The geometry optimizations showed that all atoms in the doped GDYs are still in a plane like the perfect GDY. Then, AIMD simulations at 1000, 1500, and 2000 K were performed to evaluate the stability of the doped GDYs. Although the structures were destroyed at 1500 and 2000 K, the doped GDYs kept stable at 1000 K with no bonds broken or produced. DOS results indicated that these doped GDYs are about 0.45 eV, which is very close to 0.46 eV of a GDY sheet. As to the capacity and kinetics, the N-, P-, and As-doped GDYs could achieve theoretical capacities of 2209, 2031, and 1681 mA h/g, respectively, much higher than 744 mA h/g of the perfect GDY. The higher capacities might be owing to the loss of acetylenic linkages. The charge density isosurfaces showed charge sharing between Li atoms and the doped GDYs. The diffusion barriers of Li atoms on the N-, P-, and As-doped GDYs are 0.36, 0.34, and 0.31 eV, which are close to that of the monolayer GDY and smaller than that of graphene. In short, this work indicates that the N-, P-, and As-doped GDYs are good anode candidates for LIBs due to their excellent capacities and kinetics.
